# Complex migration history is revealed by genetic diversity of tomato samples collected in Italy during the eighteenth and nineteenth centuries

**DOI:** 10.1038/s41438-020-0322-4

**Published:** 2020-07-01

**Authors:** M. R. Ercolano, A. Di Donato, W. Sanseverino, M. Barbella, A. De Natale, L. Frusciante

**Affiliations:** 1 Department of Agricultural Sciences, University of Naples ‘Federico II’, Portici, Italy; 2 Sequentia Biotech, Bellatera (BCN), 08193 Spain; 3 Department of Biology, University of Naples Federico II, Complesso Universitario di Monte Sant’Angelo, Via Cintia, 80126 Naples, Italy; 4 Società dei Naturalisti, Via Mezzocannone 8, 80134 Naples, Italy

**Keywords:** Plant domestication, Genome evolution, Plant breeding

## Abstract

Native to South America, the tomato is now grown almost worldwide. During its domestication and improvement, important selection signatures were fixed in certain agronomic and adaption traits. Such traits include fruit morphology, which became a major target for selection over the centuries. However, little is known about precisely when some mutations arose and how they spread through the germplasm. For instance, elongated fruit variants, originating both via mutations in SUN and OVATE genes, may have arisen prior to domestication or during tomato cultivation in Europe. To gain insights into the tomato admixture and selection pattern, the genome of two tomato herbarium specimens conserved in the Herbarium Porticense (PORUN) was sequenced. Comparison of the DNA of herbarium samples collected in Italy between 1750 and 1890 with that of living tomato accessions yielded insights into the history of tomato loci selection. Interestingly, the genotype of the more recent sample (LEO90), classified in 1890 as the oblungum variety, shows several private variants in loci implicated in fruit shape determination, also present also in wild tomato samples. In addition, LEO90, sampled in the nineteenth century, is genetically more distant from cultivated varieties than the SET17 genotype, collected in the eighteenth century, suggesting that elongated tomato varieties may originate from a cross between a landrace and a wild ancestor. Findings from our study have major implications for the understanding of tomato migration patterns and for the conservation of allelic diversity and loci recovery.

## Introduction

The evolutionary path of tomato (*Solanum lycopersicum*) has been elucidated by comparing genomes of cultivated varieties and wild species^1,2^. Tomato domestication probably occurred in the Andean region of Ecuador and Peru, and was completed in Mesoamerica^3^. Subsequently, human selection and extensive crop breeding led to conspicuous phenotypic changes in tomato. Selection sweeps promoted the diversification and genetic differentiation into fresh and processing tomato market classes^2^. Chiefly focusing on improving yield production, fruit quality, and disease resistance traits, breeding has given rise to wild species genome introgression^4^. The traits that were most likely selected during the tomato domestication were fruit morphological traits. Recently, a tomato pan-genome exploration revealed selection signatures in genes involved in important agronomic traits and several gene losses during tomato domestication and improvement^5^.

However, many questions about the events that occurred during domestication and selection processes remain unanswered. Some changes in fruit shape obtained in “modern” cultivars may have originated after the tomato was brought to Europe about 500 years ago, although it is not well understood when and where such alleles arose and how they spread through the germplasm. Multiple evolutionary processes in small cherry fruit, round large fruit, and elongated fruit have been postulated. For example, elongated accessions are evolutionary intermediates between large round and small size accessions^2^. Some mutations may have arisen prior to domestication or during the selection of cultivated tomato in Europe. Elongated tomato fruits may derive from a hybrid between large round and small size tomatoes, which, based on their distribution, originated in Europe^6^.

In recent years, several genes affecting such traits have been identified^7–9^. Xiao et al.^9^ asserted that elongated variants were obtained from SUN gene duplication. Elongated fruit may also have originated from mutations occurring in the OVATE gene^6^. A shift in cell division patterning during the development of the ovary led to the final fruit shape outcome^10^.

Although several hypotheses have been proposed, the exact geographical origin of the elongated groups has not been established^6^. The advent of ancient DNA analysis may provide important insights into the history of tomato loci selection. Previous small-scale ancient DNA studies limited to cytoplasmatic DNA helped to reveal patterns of crop adaptation and migration^11^. Whole nuclear genome sequencing allows investigation of the impact of selection events at genome-wide level. Therefore, nuclear genome-scale studies on ancient DNA have been conducted in recent years, paving the way for identification of allele frequency differences and candidate loci selection^12^. To better understand the history and spread of tomato in Italy and Europe as a whole, we sequenced the genome of two tomato herbarium specimens conserved in the Herbarium Porticense (PORUN). Whole-genome sequences of herbarium samples, collected between 1750 and 1890 in Italy by botanists Domenico Cirillo and Orazio Comes, were compared with living tomato accessions, to reveal the relationship with wild and cultivated landraces, and to investigate migration patterns of tomato in previous centuries.

## Results

### aDNA sequencing

**Fig. 1:**
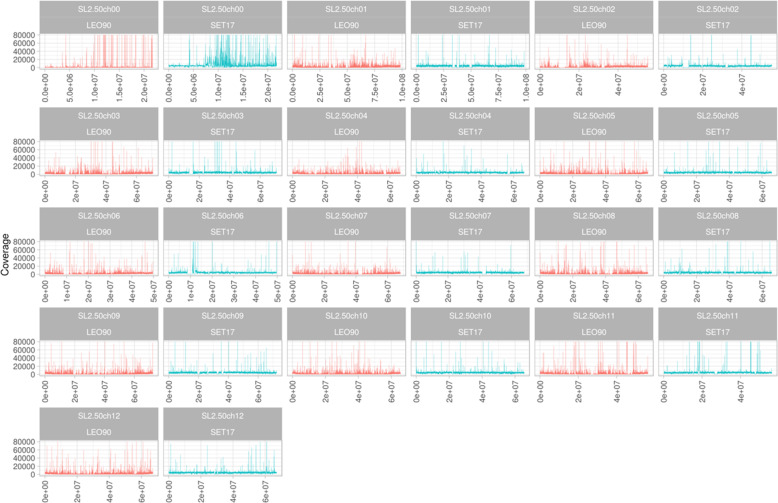
Reads distribution along chromosomes subdivided into 10 kbp bins for genotype SET17 (blue) and LEO90 (red). X-coordinate shows chromosome length. Y-coordinate shows reads coverage (range 0–100,000 in all plots)

The ancient DNA of tomato samples SET17 and LEO90 conserved in PORUN MUSA Museum (Supplementary Fig. 1) were sequenced by following a pair-end sequencing strategy. After the quality check, a total of 83,941,779 of aDNA reads were extracted from SET17, whereas 34,300,900 reads were obtained from LEO90, with a mean read length of 92.6 bp for the former sample and 80.5 bp for the latter (Supplementary Table 1). Given an expected size of about 860 Mb (SL2.50) for a tomato genome, an average coverage of about 8× and 4× genome equivalent was obtained, respectively, for SET17 and LEO90. More than 80% of LEO90 (29,102,803) and SET17 (72,655,534) reads were mapped on the reference genome of *S. lycopersicum* (genome assembly SL2.50). To obtain a more robust data set, PCR duplicates and reads with a mapping quality below 30 were removed (Supplementary Table 2). After this effort, about 3,743,080 (5.4%) reads for LEO90 and 5,005,751 (2.9%) were available for genome analysis with average coverage for covered regions of 5.5 and 3.07, respectively (Supplementary Table 3). The chromosome distribution of sequence reads reveals that, albeit with a low coverage, they are present along all chromosomes. The main peak in reads was identified on chromosome 0, suggesting that much cytoplasmatic DNA was well conserved (Fig. 1).

### DNA variome analysis

**Fig. 2:**
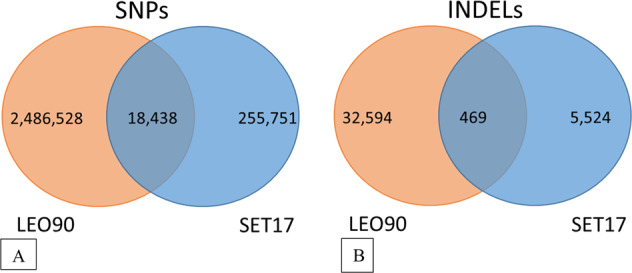
Venn diagrams of variants identified in LEO90 and SET17. (**a**) Common and private SNPs (**b**) Common and private INDELs

Overall, the sequencing of SET17 revealed the presence of 274,189 single nucleotide polymorphisms (SNPs) and 5993 small INDELs (Fig. 2). LEO90 showed almost 10 times more variants than SET, with 2,484,966 SNPs and 33,063 INDELs. A small number of variants is shared by the genotypes, corresponding to 18,438 SNPs and 469 INDELs. The variants located in important chromosome regions harboring genes putatively involved in tomato domestication and improvement sweeps^2^ are summarized in Table 1. It is worth noting that also in such regions the number of SET17 variants (15,561) was lower than that of LEO90 (254,386). In all, we identified 5585 variants in 2823 *Set17* genes and 167,659 variants in 4823 *LEO90* genes involved in domestication sweeps, as well as 3684 variants in 2127 *SET17* genes and 132,797 variants in 3722 *LEO90* genes covering improvement sweeps (Supplementary Tables 4–6). Comparing our samples with the Tomato Genome Resequencing Project (TGRP) + 3 genotype data set (Supplementary Table 7), containing tomato cultivars and related wild species data, allelic variations in one or more genotypes were highlighted. The shared improvement gene sweep variants with landrace genotypes numbered 2636 in SET17 and 9128 in LEO90 (Table 1).

**Table 1:** Number of variants in domestication and improvement sweeps

		LEO90	SET17	Common
Domestication sweeps	Variants in region	254,386	15,561	938
	Variants in genes	167,659	5,585	549
	Genes with variants	4823	2823	214
	Shared variants with landrace genotypes from 82 TGRP + 3^a^	11,489	4632	343
Improvement sweeps	Variants in region	225,245	10,775	766
	Variants in genes	132,797	3684	478
	Genes with variants	3722	2127	153
	Shared variants with landrace genotypes from 82 TGRP + 3^a^	9128	2636	324

Sequence variants were identified from 2000 bp upstream to 2000 bp downstream of the gene

^a^82 TGRP + 3 = genotypes

### Phenotypic analysis and detection of variants in target loci

The morphological traits of two dried tomato plants (SET17 and LEO90) conserved in PORUN were analyzed. Plant growth parameters showed no great difference, except in leaf attitude, whereas inflorescences differed in the number of flowers, color, style position, and shape (Supplementary Table 8). The tomato fruit type of SET17 is not reported, as no fruit diagnostic traits were revealed, whereas LEO90 showed portions of ellipsoid fruits (Supplementary Fig. S1).

**Fig. 3:**
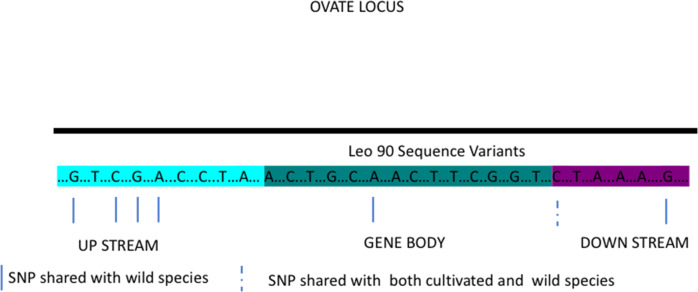
Graphical representation of OVATE locus. Non-synonymous SNPs identified in sampled LEO90, shared with wild species are indicated by blue lines and shared with both cultivated and wild species by dashed lines

More than 100 gene loci involved in the determination of tomato morphological traits were assessed for polymorphism. A high percentage of genes belonging to all investigated classes showed variants. However, the total number of varied genes is not indicative of the specificity of variants for *LEO90* or *SET17* genes. The two genotypes share only 21 variants. On average, 290 variations per trait, ranging from 0 to 689, were identified in LEO90 and seven variations, ranging from 1 to 12, in SET17 (Table 2). In LEO90, a considerable number of variants were found in fruit size genes (*Fw2.2* and *Fw2.3*) and in genes involved in fruit shape determination (*FAS LC* and *OVATE*). The genes related to fruit shape in LEO90 showed a higher polymorphism and a larger number of SNPs shared with wild species. The variants identified in LEO90 in the OVATE locus shared with wild species (Supplementary Table 9) are shown in Fig. 3.

**Table 2:** Number of variants identified in tomato loci related to morphological traits

Trait	Loci analyzed per trait	Common variants	Private SET17 variants	Private LEO90 variants
Fruit color	18	5	12	689
Fruit shape/size	72	16	15	670
Fruit weight	2	0	1	69
Pericarp thickness	2	0	2	0
Plant architecture	8	0	6	29
Total	102	21	36	1457

### Phylogenetic analysis and principal component analysis

**Fig. 4:**
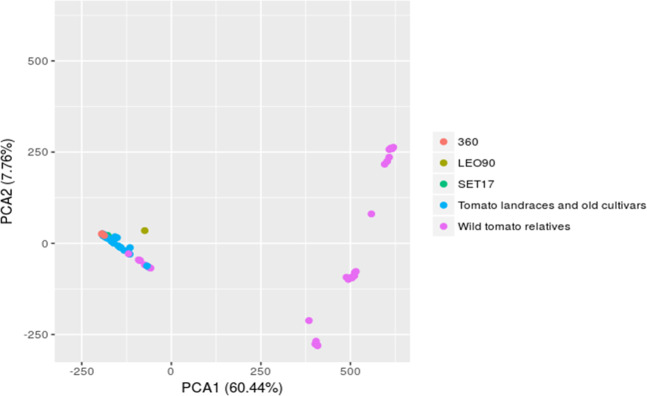
Principal component analysis (PCA) based on SNPs identified in herbarium, wild and cultivated tomato samples. SNPs identified in SET17 and LEO90 genomes were compared to the SNPs identified in wild and cultivated tomato accessions

To explore the pedigree of LEO90 and SET17, we compared their genomes with a panel of previously sequenced wild and cultivated tomato genomes^1,2^. Principal component analysis (PCA) was performed using the above data set (Fig. 4). The graphical pattern of the first two principal components (PCs) shows a group with a clear edge (positive PC2) corresponding to cultivated species, including SET17 sample. The closest genotype to SET17 was found in accession 031 (Supplementary Fig. 2), collected in an area close to Naples. Interestingly, few wild genotypes belonging to the species *Solanum pimpinellifolium*, *Solanum galapagense*, and *Solanum cheesmaniae* cluster on the opposite edge of the group (negative PC1 and PC2). By contrast, the PCA plot revealed that LEO90 is not included in the cultivated varieties group even if it is quite close to such accessions. The rest of the wild accessions are arranged along the PC2 component in a separate group, confirming the fundamental patterns of population structure across present-day accessions and wild species reported in previous studies. A PCA performed only on the cultivated accession data set supported the finding that the LE090 is not closely related to cultivated samples (Supplementary Fig. 3).

## Discussion

To shed light on the history of the spread of tomato in Europe, the draft genomes of two *S. lycopersicum* (formerly *Lycopersicon esculentum*) samples stored in PORUN were investigated. Detailed analysis of herbarium cards, including handwritten labels and notes, yielded insights into the significance of the PORUN collection samples within their historical and scientific context. The older sequenced sample belongs to the Cirillo Collection, a famous private herbarium from the eighteenth century. Cirillo’s studies focused both on wild plants and on plants cultivated in his garden^13,14^. The herbarium bears one of Cirillo’s handwritten cards^15^, confirming that the specimen is one of the oldest herbarium tomato samples preserved in Italy. The tomato sample, however, does not possess other indications, except for taxonomic data. The date is unknown, but may undoubtedly be attributed to the second half of the eighteenth century when Cirillo was active. The second sample used for DNA analysis belongs to the Comes Collection (nineteenth to twentieth centuries) but is currently conserved in the General Herbarium Collection^16^. Comes’ dried herbarium sample is accompanied by a handwritten card with annotation of the species (*Lycopersicum esculentum* Mill. var. *oblungum*), the location of the collection (Hortus Botanicus Porticense), and the collection date 23 June 1890. The sample leaves, traces of flowers, and elongated fruits supported specimen and fruit type assignment. Assessment of the morphological traits of herbarium plant samples reveals that they are fully similar to modern cultivated tomato varieties, except for stigma position and size. According to Peralta et al.^17^, tomato domestication and improvement have been marked by changes in style traits. The history of the herbarium collections is completely different and the tomato samples preserved may come from different geographic areas.

The aDNA extracted from the above samples allowed next generation sequencing (NGS) reads of good quality to be obtained even if most of them map on chromosome 0. The above chromosome is an artifact that groups unmapped scaffold and cytoplasmatic DNA, supporting the finding that mitochondrial or plastid DNA is more easily retrieved in ancient specimens than is nuclear DNA^18,19^. Therefore, the bias in fragment amplification of mitochondria or chloroplasts DNA should be taken into consideration. In our experiment, despite the inherent difficulty in targeting short fragments of nuclear DNA, the distribution of reads along chromosomes was sufficient to cover regions containing important genomic loci.

Obtaining nuclear genomic sequences is helpful for the acquisition of knowledge about gene family variability. To this end, a suite of tomato genomes from several repositories was used to compare allelic diversity and to expand knowledge concerning tomato migration routes and exploitation. A large number of variants in genes affecting loci regulating domestication and improvement traits were discovered, most being supported by variants found in modern accessions. In the literature most tomato fruit variability is explained by the *Fw2*, *Fw3*, *FAS*, *SUN*, *OVATE*, and *LC* genes^1^. In our samples, the SNPs in fruit morphological traits were explored further, to classify variant type and position. Interestingly, the LEO90 genotype, classified in 1890 as belonging to the *oblungum* variety, shows several private variants in loci implicated in fruit shape determination, present also in wild tomato samples. In contrast, the few detected variants in SET17 are more similar to those found in modern cultivated varieties.

The PCA analysis conducted on our SNP data set in comparison with public released data sets highlighted the high similarity of SET17 with a genome of tomato landraces (Accession 031; Supplementary Table 7) obtained from the same area as the herbarium collection. Accession 031, harvested in the region of Campania at the beginning of the last century, proved very similar to the SET17 sample, supporting the hypothesis that the cultivation of tomato in the eighteenth century was all over the place^20^. Rapid evolution of traits under human selection led to conspicuous phenotypic changes and adaptations to several environments^5^. A high selection pressure in fruit morphological traits occurred during early selection of tomato in Europe^2,21^. The Italians were considered the foremost leaders in the selection of tomato varieties in the eighteenth and nineteenth centuries^20^, and the above finding suggests that major agronomic traits were already available in Italian varieties at the time.

The LEO90 specimen was not included in the group of cultivated accessions, suggesting that elongated tomato varieties may have originated from a cross between a landrace and a wild ancestor. According to legend, seeds of the San Marzano elongated variety were a gift from the King of Peru to the King of Naples during the mid-1700s. This defies logic, as there was no king of Peru at the time, but it seems plausible that a natural cross with a South America native accession originated an oblong fruit variety. Tomatoes containing OVATE mutated alleles may have been shipped to Europe centuries after the delivery of the first tomato, performing mutations LC and FAS^6^. Trade in plants and food between Italy, Spain, and America was quite common^22^. In addition, Comes had several botanical exchanges with South American countries^16^ and may have received seeds of an oblong tomato variety. It is worth noting that Comes cataloged the herbarium sample as “*Lycopersicum esculentum* var. *oblungum*,” collecting also the fruits, indicating that the trait in question might be important, or indeed unique.

The pattern of genetic diversity, population structure, and introgression could have played a major role in the allele frequency of the observed variety. Tomato with elongated fruits sampled in 1890 was genetically less close to cultivated varieties than the sample collected in the eighteenth century. Many tomato species show admixture due to natural hybridization^23^ and outcrossing rates are highly variable and related also to population structure and climate^24,25^. Tomato experienced intermediate stages during its domestication such as hybridization with ancestral populations. Many traits, reported as typical of cultivated tomatoes predated domestication, were lost and reselected in cultivated tomato^26^.

The introduction of novel alleles can be fixed by selection, leading to introgression of one species’ alleles into the background genome of another. Findings from our study could have major implications for the understanding of tomato variation that occurred in Europe between the eighteenth and nineteenth centuries and for the conservation of allelic diversity. To this end, the ten *L. esculentum* exsiccata stored in the Historical Herbarium of Portici (PORUN, MUSA Museum of the University of Naples Federico II), collected between the 1750 and the 1892 could be further investigated at genomic level to underpin undetected diversity. Interestingly, genome engineering strategies enabled the recovery of target wild sweep loci and de novo domestication of *S. pimpinellifolium*. In particular, the editing of the *OVATE* gene and other loci altering the shape and size was promoted by CRISPR/Cas9. Loss of the OVATE gene function in both alleles resulted in elongated fruit shape^6,27^. The genetic diversity discovered in old varieties in our study could be exploited for breeding purposes using site-specific mutagenesis approaches.

## Material and methods

We selected two *L. esculentum* specimens stored in the PORUN, MUSA Museum (http://www.centromusa.it/it/), University of Naples Federico II (Supplementary Fig. 1). From each sample, we selected a part of dried leaf where the removal of fragments would not significantly compromise its overall appearance and value. The first specimen, referred to as SET17, according to the label reporting information related to the species and the identity of the collector, was dated around 1750, as it was collected in the eighteenth century in the historical herbaria of the Neapolitan botanist Domenico Cirillo^15^; at the time, it was labeled as “*Solanum* (*Lycopersicon*).” The second specimen, referred to as LEO90, was part of the personal collection of the botanist Orazio Comes^16^, dated 1890 and labeled as “*Lycopersicum esculentum* Mill. var. *oblungum*”. Data about the origin and extent of natural variability present in the collections were also recorded, as herbaria typically contain multiple specimens collected by a botanist in the same area, which can yield useful information^15,16^.

### DNA extraction and quality check

Total genomic DNA was isolated from herbarium leaves dated between 1750 and 1890 following the Ames and Spooner^11^ protocol with slight modifications*.* A pair of primers trn V/ndh C fw (F: 5′-AG TTT ACT CAC GGCAAT CG-3′) and trn V/ndh C rev (R: 5′-GGA GGG GTT TTT CTT GGT TG-3′) were used to perform PCR reactions with 10 ng of DNA, 10 pmol primers, 1 U of Taq DNA polymerase kit (Invitrogen, Carlsbad, CA, USA), 10 pmol dNTPs, and 2 mM MgCl_2_ in 25 μl reaction volumes. Amplification was performed using the following cycling conditions: 1 min at 94 °C, followed by 30 cycles of 1 min at 94 °C, 1 min 30 s at 60 °C, and 2 min at 72 °C, with a final extension for 7 min at 72 °C. All positive controls were manipulated separately from the herbarium samples to avoid contamination using the same master mix. Amplicons were separated by electrophoresis on agarose gel (1.5%) and photographed by a Gel Doc system (Bio-Rad, Milan, Italy). They were sequenced using the BigDye Terminator Cycle Sequencing Kit (Applied Biosystems, Foster City, CA, USA) and run on automated DNA sequencers (ABI PRISM 3100 DNASequencer, Applied Biosystems). ClustalW (www.genome.jp/tools/clustalw) was used to align sequence data with corresponding reference sequences (data not shown).

### Preparation of library and sequencing

The genome library for sequencing the two DNA samples was prepared using the Illumina Nextera XT DNA sample preparation kit (Illumina, San Diego, USA) according to the manufacturer’s protocol. The gDNA was fragmented by random transposon integration. DNA adapters with sample-specific barcodes were added to each sample before PCR amplification. The library was size-selected using magnetic beads and the paired-end sequencing of samples was conducted on the Illumina HiSeq 2500 (Illumina, San Diego, USA) at Base Clear facilities (Einsteinweg 5, 2333 CC Leiden, The Netherlands). FASTQ sequence files were obtained using the Illumina Casava pipeline version 1.8.3. Initial quality assessment was performed using the Illumina Chastity filtering tool. Subsequently, reads containing adapters and/or PhiX control signals were removed using an in-house filtering protocol. The second quality assessment was based on the remaining reads using the FASTQC quality-control tool version 0.10.0. Adapters were removed from raw sequencing data, preserving the longest high-quality part of the NGS read (Supplementary Table 1) with a minimum length of 35 bp and a minimum PHRED quality score of 25.

### Data processing and SNP calling

The data from sequencing were processed using the Super-W pipeline (http://www.sequentiabiotech.com/sequentia-research-and-development/projects/). The pipeline was divided into three steps: filtering, mapping, and variation calling. After the filtering step, all the samples were mapped against a *S. lycopersicum* genome v.2.50 (https://solgenomics.net) with BWA version 0.7.12-r1039^28^ using the bwa aln algorithm. The mapped files were filtered for removing PCR duplicates and reads with a mapping quality below 30 (probability < 99.9% of being the true alignment) using Picard (http://broadinstitute.github.io/picard/), compressed in bam files, sorted, and indexed^28^, creating as output a bam file and a statistical output with all the information about the trimming and mapping. The variant calling (SNPs) and short deletion and insertion polymorphisms (DIPs) was performed with SAMtools^28^ through a double calling step without filtering threshold. The first run of SAMtools was used to perform a multiple pileup (Mpileup), in which all samples were used together to perform the SNP and DIP calling, whereas a second run was used to call small variations independently for each sample. The final result of the two previous analyses were compared with the variant data of cultivated and wild tomato listed in Supplementary Table 7, stored in TGRP repository and at Sequence Read Archive (SRA) archive SRP045767 by Aflitos et al.^1^ and Lin et al.^2^, respectively.

### Construction of the phylogenetic tree and PCA analysis

Sequence data from our samples and from the TGRP and at SRA archive SRP045767 were combined (TGRP + 3 data set) to build a phylogenetic tree and to perform PCA analysis. The variant calling results were converted into Boolean format, i.e., for each observed SNP, 0 or 1 was assigned to each genotype for the absence or presence, respectively. The matrix obtained was imported in R and analyzed with the ape package^29^ to produce a neighbor-joining tree (“nj” command) and to perform PCA analysis, with the function “prcomp.” The PCA was then plotted with the ggplot2 package^30^.

#### Data deposition

Raw sequence data from SET17 and LEO90 supporting the findings of this study are available at the National Center for Biotechnology under SRA accession PRJNA592130. Pipelines and Scripts used in this work are available at Source forge site: https://sourceforge.net/projects/superw/.

## Supplementary information

Supplementary file 1

Supplementary file 2
