# Supplementary material for: Complex migration history is revealed by genetic diversity of tomato samples collected in Italy during the eighteenth and nineteenth centuries
Source: Hortic Res. 2020 Jul 1;7:100. doi: 10.1038/s41438-020-0322-4 (PMC7327043; doi:10.1038/s41438-020-0322-4)

Supplementary information

This file includes a picture of herbarium samples LEO90 and SET17 (Figure S1) sequenced in this work (Figure S1), a neighbor-joining tree reporting the estimated phylogenetic relationships among wild and cultivated accessions analyzed in this study (Figure S2) and a Principal component analysis performed on S. lycopersicum samples (FigureS3).

Figure S1. Herbarium samples, LEO90 (a) and SET17 (b)

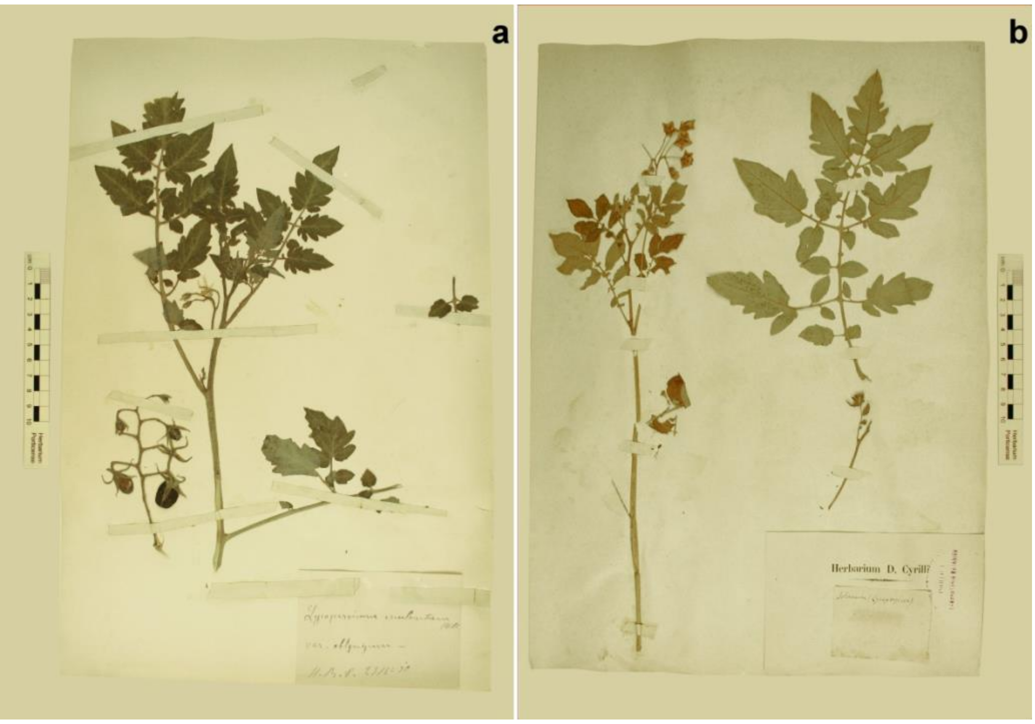

Figure S2. The neighbor-joining tree showing the phylogenetic relationship among Solanum spp. accessions. Labels with the bootstrap values are reported above the branches. Green spots indicated cultivated tomato accessions, yellow spot wild *Solanum* species, red spot LEO90 and SET17 samples sequenced in this study.

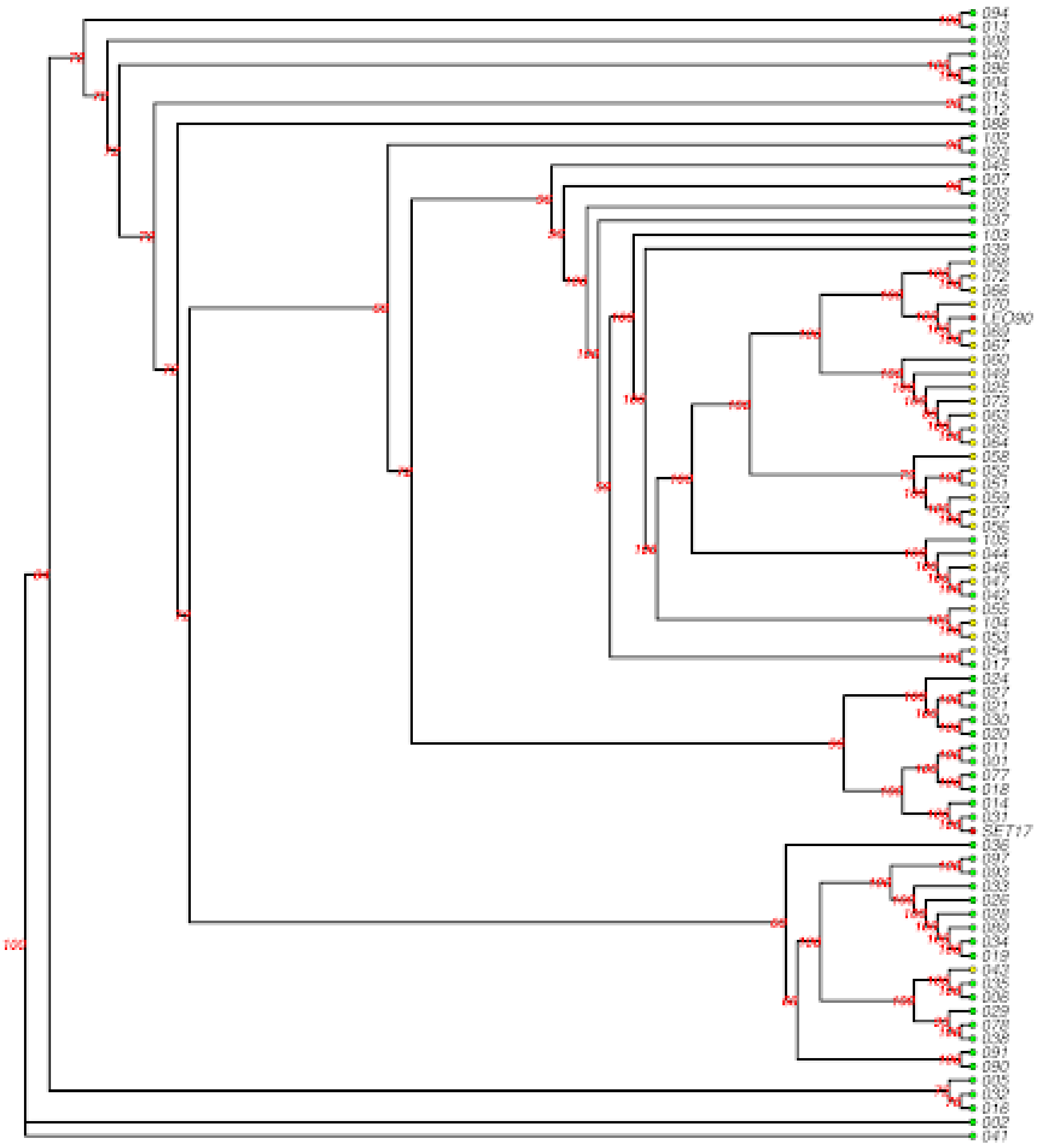

Figure S3. Principal component analysis (PCA) performed on SNPs identified in SET17 and LEO90 compared to tomato old cultivars and landraces

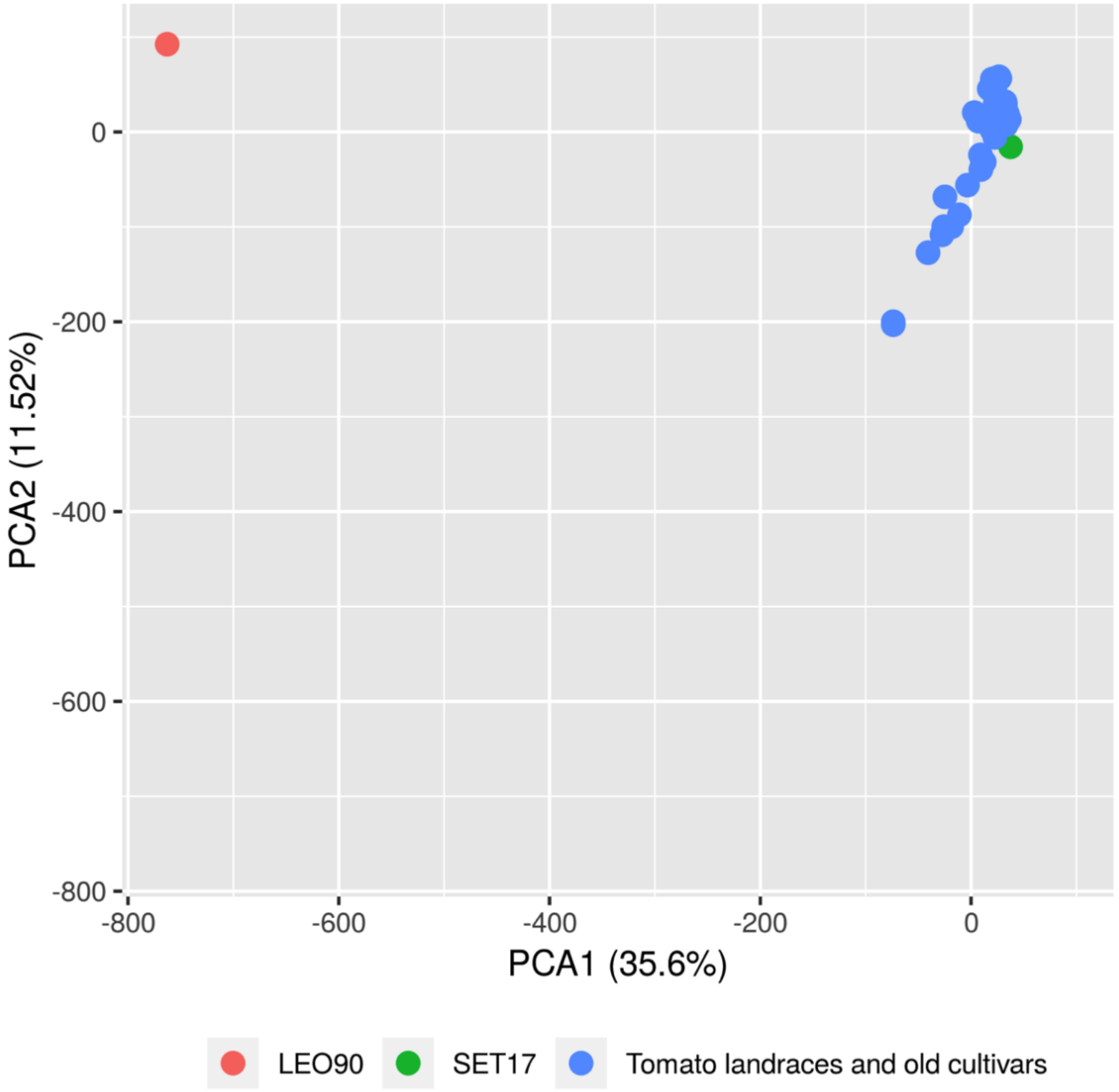

Supplement: Supplementary file 1 [file 41438_2020_322_MOESM1_ESM.pdf]
